# The putative β-glucosidase BGL3I regulates cellulase induction in *Trichoderma reesei*

**DOI:** 10.1186/s13068-018-1314-6

**Published:** 2018-11-19

**Authors:** Gen Zou, Yanping Jiang, Rui Liu, Zhihua Zhu, Zhihua Zhou

**Affiliations:** 1 CAS-Key Laboratory of Synthetic Biology, CAS Center for Excellence in Molecular Plant Sciences, Institute of Plant Physiology and Ecology, Shanghai Institutes for Biological Sciences, Chinese Academy of Sciences, 300 Fenglin Rd, Shanghai, 200032 China; 2 Shanghai Key Laboratory of Agricultural Genetics and Breeding; Institute of Edible Fungi, Shanghai Academy of Agriculture Science, 1000 Jinqi Rd, Fengxian, 201403 Shanghai China; 3 Southwest Jiaotong University, Chengdu, 611756 Sichuan China

**Keywords:** β-Glucosidase, Cellulase, Induction, Regulation, *Trichoderma reesei*

## Abstract

**Background:**

The filamentous fungus *Trichoderma reesei* (anamorph of *Hypocrea jecorina*) displays increased cellulase expression while growing on inducers such as lactose or cellulose. However, the mechanism of cellulase induction in *T. reesei* is not yet completely characterized. Here, a protein annotated as β-glucosidase (BGL3I) was found to be involved in cellulase induction in *T. reesei*. The effects of BGL3I on cellulase production have not yet been fully understood.

**Results:**

Deletion of the *bgl3i* gene had no influence on the growth of *T. reesei*, but significantly increased its cellulase activities. Deletion of *bgl3i* also resulted in decreased extracellular galactosidase activity, but significantly increased transcription of lactose permeases, which might be involved in lactose transport. Furthermore, deletion of *bgl3i* enhanced the transcription levels of intracellular β-glucosidases *cel1a, cel1b* and the regulator *xyr1*, which are all essential for lactose induction in *T. reesei*. BGL3I was found to have a relatively high ability to hydrolyze sophorose, which is proposed to be the strongest natural inducer of cellulase synthesis in *T. reesei*.

**Conclusions:**

BGL3I may take part in the complex regulating system of cellulase induction. The deletion of *bgl3i* offers a new strategy to improve *T. reesei* strain performance.

**Electronic supplementary material:**

The online version of this article (10.1186/s13068-018-1314-6) contains supplementary material, which is available to authorized users.

## Background

Plant biomass, which represents the most abundant renewable carbon source on the earth, can be converted into environmentally friendly energy and chemicals; thus, cost-effective and economical bioconversion is essential [1]. The filamentous fungi *Trichoderma reesei* (teleomorph *Hypocrea jecorina*) is one of the most well-known industrial strains producing cellulase and hemicellulase, which releases fermentable soluble sugars from plant biomass. The high production levels of lignocellulose-degrading enzymes in *T. reesei* are dependent on induction by cellulose- and hemicellulose-containing plant polysaccharide mixtures. Disaccharide lactose (1,4-*O*-β-d-galactopyranosyl-d-glucose) also serves as an inducer of cellulase formation in *T. reesei* and is presently the only soluble substrate that can be applied economically for this purpose [2, 3]. Approximately, 1.2 million tons of disaccharide lactose is produced every year worldwide from whey processing industries or cheese manufacturing. The solubility of lactose is favorable for manipulation and enzyme separation such that lactose is preferred over cellulose as an inducing substrate for cellulase production [4].

There are two principal pathways to make use of lactose in fungi. The first is extracellular hydrolysis of lactose into d-galactose and d-glucose by β-galactosidases, followed by uptake of the generating monosaccharides by respective permeases; and the second is the uptake of the lactose itself followed by intracellular hydrolysis [3]. Previously, it was considered that only the first pathway existed in *T. reesei* based on the absence of lactose permease orthologues or intracellular β-galactosidases in the genome of *T. reesei*, and the absence of intracellular β-galactosidase activity on lactose in *T. reesei* [3, 5]. Neither d-galactose nor d-glucose, or mixtures of the two, can induce transcription of cellulase genes such as lactose. However, the constitutive overexpression of extracellular β-galactosidase *bga1* abolishes the induction of cellulase expression by lactose [5–7]. Interestingly, three major facilitator superfamily transporters were recently identified as lactose permeases and are essential for cellulase induction by lactose in *T. reesei* [2, 8, 9]. Conversely, the disruption of the intracellular β-glucosidases CEL1A and CEL1B compromises the induction of *cbh1* gene expression on lactose medium, which shows that β-glucosidases play an important role in cellulase induction by using lactose as inducer [4]. However, except for the widely studied extracellular β-glucosidase CEL3A (BGLI) and intracellular β-glucosidases CEL1A (BGLII) and CEL1B, there are still eight other β-glucosidase genes predicted in the genome of *T. reesei* which have not been studied well [10].

Unlike the previously reported studies [11], our recent study indicated that most of these β-glucosidase genes were upregulated by Xyr1 in the inducing medium (wheat bran and Avicel); however, a β-glucosidase gene named *bgl3i* (*cel3g*) was downregulated [12]. On the contrary, the expression level of *bgl3i* was upregulated in a *xyr1* deletion strain [12]. Recently, the enzyme activities of the heterologously expressed BGL3I from *Aspergillus oryzae* to different substrates have been compared with other β-glucosidases of *T. reesei* [18]. No further investigation of its potential function in cellulase induction of *T. reesei* has been carried out until now, possibly due to its lower transcription level. We are interested in why *bgl3i* reacted differently to the *xyr1* deletion than other *T. reesei* β-glucosidase genes. In this study, the non-transcription factor BGL3I was found to be involved in cellulase induction in *T. reesei* on lactose, which is the most economical and efficient inducer of cellulase production. The transcription levels of intracellular β-glucosidases *cel1a* and *cel1b* genes, as well as the positive TF *xyr1*, were significantly enhanced by the absence of the *bgl3i* gene. Further investigation demonstrated that protein BGL3I regulates cellulase induction by affecting the uptake of extracellular lactose and the hydrolysis of inducer sophorose. Based on the experimental data, it is possible that *bgl3i* plays a role in the regulation of cellulase production.

## Results

### Deletion of the *bgl3i* gene enhances cellulase induction of *T. reesei*

**Fig. 1:**
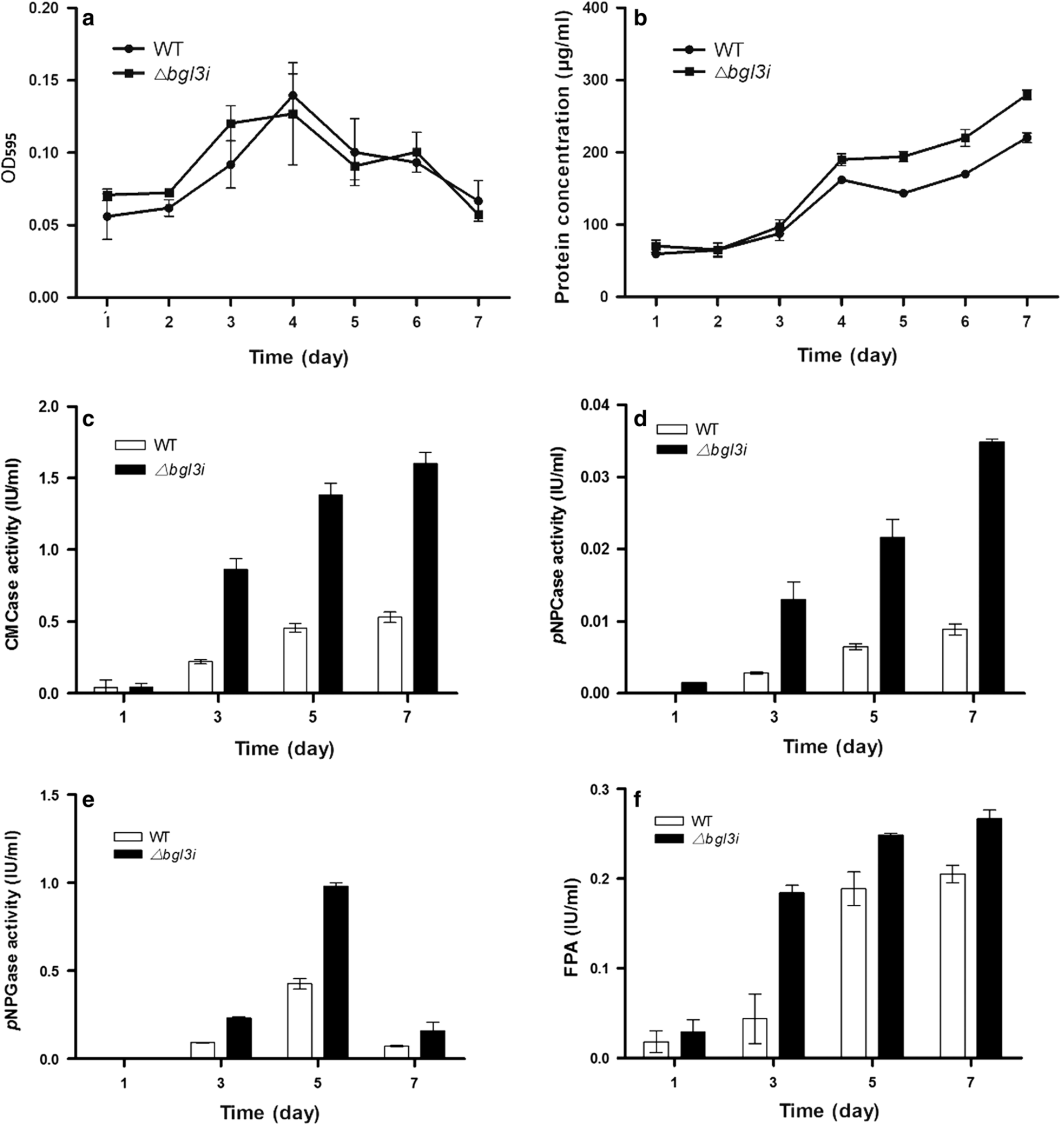
Growth, extracellular protein concentration, and cellulase formation of *T. reesei*. **a** Biomass formation, **b** extracellular protein concentration, **c** CMCase activity, **d** *p*NPCase activity, **e** *p*NPGase activity, **f** FPA of WT and Δ*bgl3i* mutant on 1% lactose. Vertical bars indicate SD and each reaction was done in triplicate

The *bgl3i* gene is composed of four exons and three introns, as predicted in JGI (Transcript ID: TRIRE2_47268), and belongs to the GH3 family according to the Pfam database. It was screened using the expression profile data of our laboratory, based on the significantly increased transcription in the *xyr1* disrupted strain compared with the WT under induction conditions [12]. The expression profile data and the cDNA sequence of *bgl3i* showed that the sites of its start codons and exon/intron junctions were not properly located on the JGI website, but were correct on NCBI (accession no. BAP59015) [12]. It is well known that the expression levels of most cellulase and hemicellulase genes are dramatically reduced in the absence of *xyr1* in *T. reesei* [13]; thus, the increased transcription of *bgl3i* in *xyr1* deletion strain is very distinctive. Therefore, to explain the difference, we generated a *bgl3i* deletion strain by replacing the *bgl3i* coding region with the *Penicillium oxalicum ura5* gene in the *T. reesei* uridine auxotrophic strain QmU2–3 (Additional file 1). Deletion of the *bgl3i* gene had no impact on growth, but significantly enhanced extracellular protein production and cellulase activities, as demonstrated by CMCase, *p*NPCase, *p*NPGase and FPA of the supernatants of the Δ*bgl3i* strain cultured in lactose (Fig. 1). The extracellular protein concentration and cellulase activities of a complementation strain re*bgl3i* (obtained by transforming the expression cassette of *bgl3i* into Δ*bgl3i* strain using native promoter and terminator) were restored to the same level as that in the parent strain QmU2–3 (Additional file 2). Also, the *bgl3i* overexpressed strain oe*bgl3i* decreased the protein concentration of the preparation (Additional file 2). All the activities of CMCase, *p*NPGase, *p*NPCase and FPA of the enzyme preparation of the oe*bgl3i* strain were decreased significantly (*P *< 0.01), compared with that of QmU2–3 (Additional file 2). Furthermore, when the Δ*bgl3i* strain was cultured in the inducers cellobiose or Avicel, as well as in repressor glucose, all of its extracellular protein production and cellulase activities, as represented by CMCase, *p*NPCase, *p*NPGase and FPA, increased significantly compared with those of the parent strain QmU2–3 (Additional file 3). All of the measured enzyme activities were increased approximately twofold, especially in Avicel. These results demonstrated that BGL3I plays an important role in cellulase production with lactose, as well as other carbon sources.

### BGL3I affects the transcription levels of the main cellulase and regulator genes

**Fig. 2:**
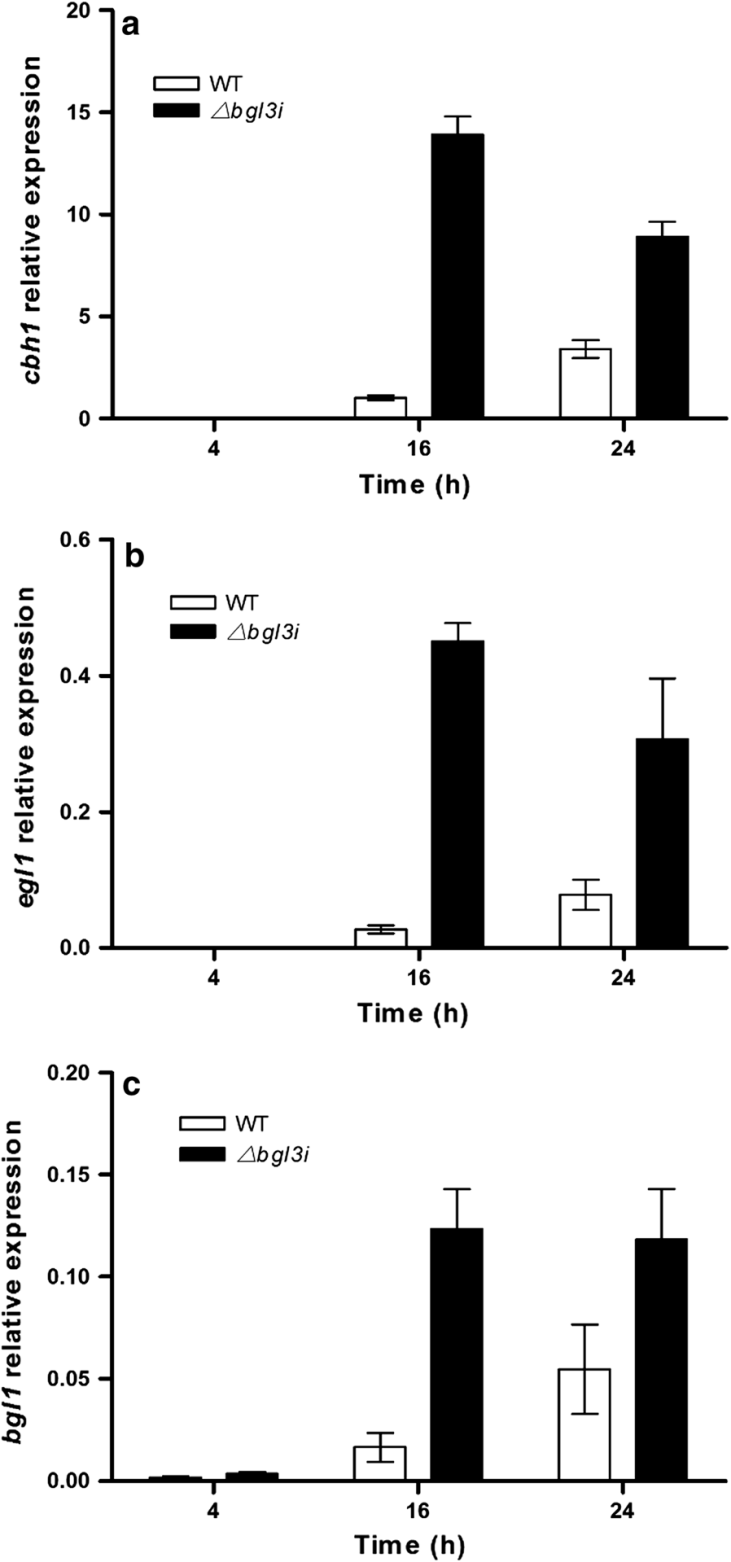
Transcription levels of the major cellulase genes. Cellulase genes were detected at 4 h, 16 h, and 24 h after the beginning of induction with 1% lactose in WT and Δ*bgl3i* mutant. **a** *cbh1* relative transcription level, **b** *egl1* relative transcription level, **c** *bgl1* relative transcription level. Transcripts were normalized to the housekeeping gene *actin*. Vertical bars indicate SD and each reaction was done in triplicate

To investigate whether and how the deletion of *bgl3i* affects the transcription level of cellulase genes, as well as related TFs, quantitative RT-PCR analysis was performed for the main cellulase genes, the genes encoding intracellular β-glucosidases CEL1A and CEL1B, as well as the cellulase TF genes, which are the essential activators of the cellulase genes expressed in response to cellulose or lactose. In accordance with the enzyme profile, investigation of mRNA by quantitative RT-PCR showed that the transcription level of the main cellulase genes (including *cbh1*, *egl1* and *bgl1*) were higher in the Δ*bgl3i* strain than that in the parent strain during culture in lactose (Fig. 2).

**Fig. 3:**
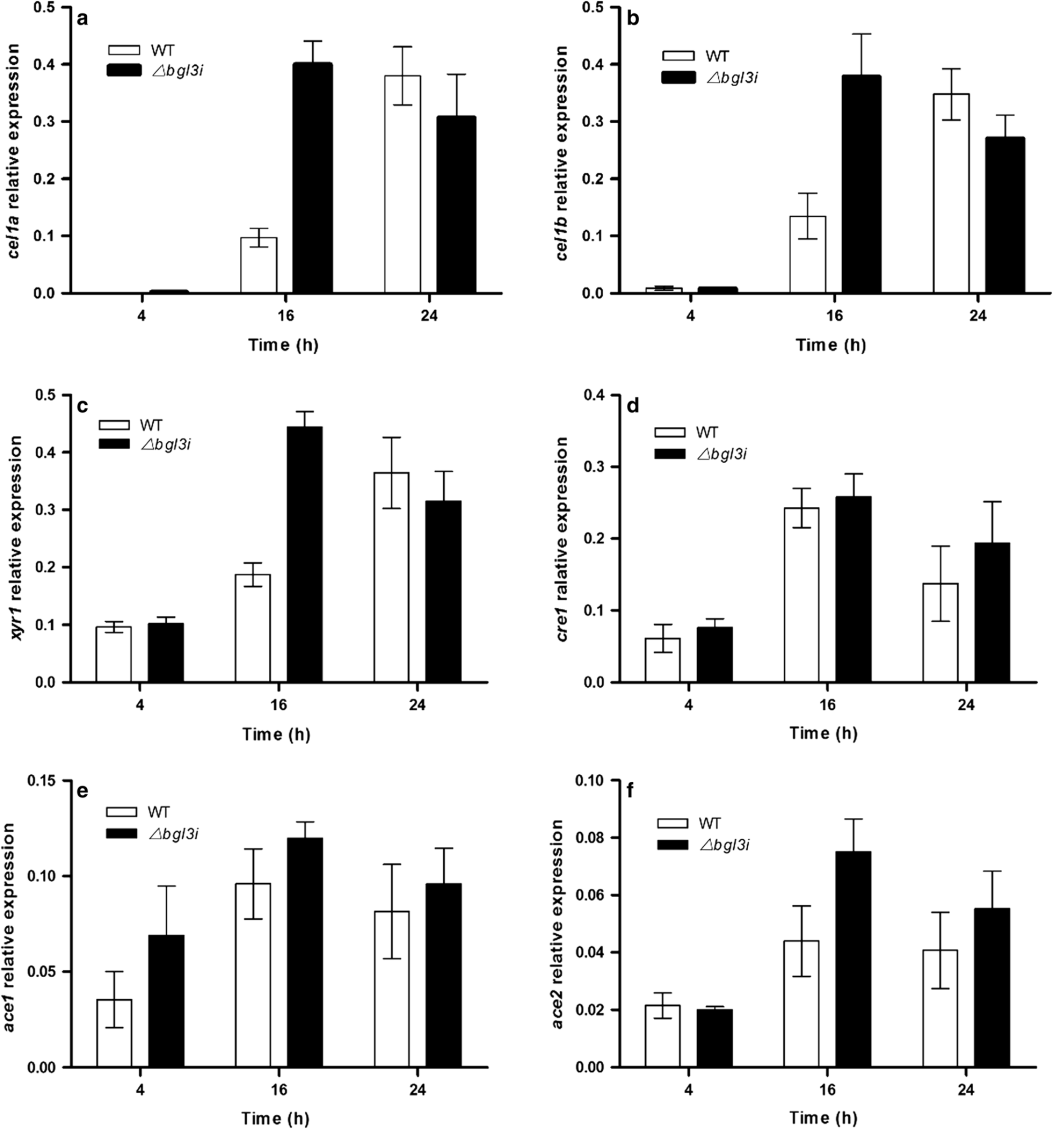
Analysis of transcription levels of intracellular β-glucosidase genes *cel1a* and *cel1b* as well as transcription factors. **a** Intracellular β-glucosidase genes *cel1a*, **b** intracellular β-glucosidases gene *cel1b*, **c** transcription factor *xyr1*, **d** transcription factor *cre1*, (E) transcription factor *ace1*, **f** transcription factor *ace2*. They were detected at 4 h, 16 h and 24 h after the beginning of the cultivation with 1% lactose in WT and Δ*bgl3i* mutant. Transcripts were normalized to the housekeeping gene *actin*. Vertical bars indicate SD and each reaction was done in triplicate

It has been reported that the intracellular β-glucosidases CEL1A and CEL1B are indispensable for the cellulase synthesis induced by lactose, which probably further results in the synthesis of Xyr1 to efficiently initiate cellulase induction in *T. reesei* [4]. In the Δ*bgl3i* strain, the transcription levels of intracellular β-glucosidase CEL1A and CEL1B were greatly elevated at 16 h after induction with 1% lactose. The transcription level of CEL1A was increased more than threefold compared with that of the parent strain QmU2–3 (Fig. 3).

For TFs involved in cellulase synthesis, the transcription levels of activators *xyr1* and *ace2* were significantly increased to 2.37- and 1.82-fold in Δ*bgl3i*, respectively, compared with that of QmU2–3. In contrast, the negative regulators *cre1* and *ace1* had similar transcription levels in both strains (Fig. 3). These results imply that BGL3I represses cellulase induction, which is initiated from CEL1A and CEL1B in response to lactose by downregulating the transcription level of CEL1A and CEL1B, and then that of Xyr1, which represents a possible pathway for cellulase induction.

Furthermore, the transcription levels of the main cellulase genes (Additional file 4) and *xyr1* (Additional file 5) in the Δ*bgl3i* strain were increased more significantly in the presence of Avicel than lactose. However, the transcription levels of *ace2* (Additional file 5) showed no significant difference between the Δ*bgl3i* strain and parent strain cultured in Avicel (*P *= 0.73). These data show that deletion of *bgl3i* caused the de-repression of cellulase induction, but the mechanism involved in the induction might be different between carbon sources. It is necessary to carry out more detailed investigation of how BGL3I is involved in the repression of cellulase induction in *T. reesei*.

### BGL3I is involved in the regulation of transportation and consumption of extracellular lactose

**Fig. 4:**
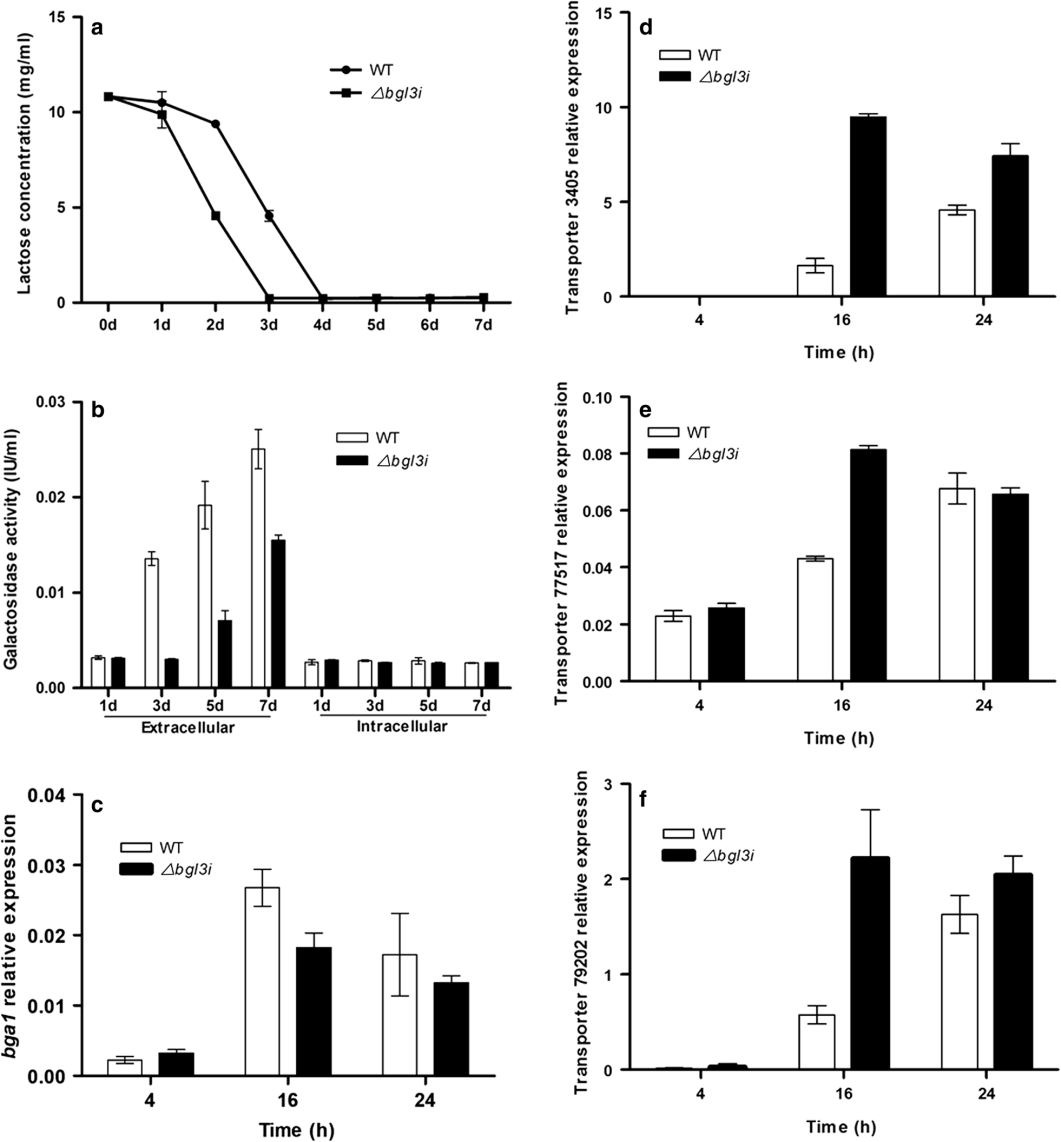
BGL3I impacted on the hydrolysis and uptake of extracellular lactose. **a** Lactose concentration, **b** galactosidase activity, **c** transcription level of galactosidase gene *bga1*, **d** transcription level of lactose permease gene 3405, **e** transcription level of lactose permease gene 77517, **f** transcription level of lactose permease gene 79202. Lactose permease genes were detected at 4 h, 16 h and 24 h after the induction with 1% lactose in WT and Δ*bgl3i* mutant. Transcripts were normalized to the housekeeping gene *actin*. Vertical bars indicate SD and each reaction was done in triplicate

The analysis of CMCase activities showed that the reducing sugar concentration of the blank control from the Δ*bgl3i* strain supernatant was obviously lower than that from the parent strain. Therefore, we set up a method to measure the lactose concentrations in the supernatants of the Δ*bgl3i* and the parent strain, based on the DNS method with lactose as the standard. As expected, the extracellular lactose concentration was significantly decreased in the supernatants of the Δ*bgl3i* strain compared with that of the parent strain after 2 days of cultivation with lactose. The lactose in the culture of the Δ*bgl3i* strain was almost exhausted within 3 days, which was a day earlier than QmU2–3 (Fig. 4a), suggesting that the Δ*bgl3i* strain may consume the extracellular lactose in the medium more quickly than the parent strain.

Regarding extracellular lactose metabolism in *T. reesei*, it is now thought that lactose can either be hydrolyzed by extracellular β-galactosidase or directly transported into the cell by lactose permeases, and then hydrolyzed by intracellular β-galactosidase [2–4, 9]. Based on these possibilities, measurement of both extracellular and intracellular β-galactosidase activity, as well as lactose permease expression analysis, was carried out. The activity of extracellular galactosidase of the Δ*bgl3i* strain was dramatically reduced to merely 22% that of the parent strain after 3 days of cultivation on lactose and remained lower than the WT during the whole fermentation process (Fig. 4b). The expression level of the main extracellular galactosidase *bga1* was also decreased after deletion of *bgl3i* (Fig. 4c). However, after measuring the galactosidase activity of BGL3I itself, it was found that BGL3I had nearly no galactosidase activity, with values 0.02 ± 0.00 U/mg toward lactose and 0.09 ± 0.01 U/ml toward ONPG (Table 1). In addition, its low expression level suggests that the higher extracellular galactosidase in the wild strain is not a result of BGL3I activity itself. Alternatively, the higher consumption rate of extracellular lactose in the Δ*bgl3i* strain may be due to a faster assimilation of extracellular lactose into cells, rather than to the faster hydrolyzation of lactose by extracellular galactosidases. Interestingly, higher expression levels of three lactose permeases involved in lactose transport were observed in the Δ*bgl3i* strain, among which lactose permease 3405 (Transcript ID: TRIRE2_3405) showed the highest increase in transcription, followed by lactose permeases 79202 (Transcript ID: TRIRE2_79202) and 77517 (Transcript ID: TRIRE2_77517) (Fig. 4d–f). The data suggest that deletion of *bgl3i* might result in efficient transport of extracellular lactose in the culture medium into the cell via lactose permeases, followed by initiation of cellulase induction.

**Table 1:** Enzymatic characterization of recombinant β-glucosidases

BGLs	Specific activity (U/mg)								
	G2	G3	G4	G5	Lactose	Sophorose	*p*NPG	Salicin	ONPG
BGL3I	0.09 ± 0.01	0.06 ± 0.01	0.04 ± 0.00	0.01 ± 0.00	0.02 ± 0.00	4.12 ± 0.35	2.98 ± 0.16	0.43 ± 0.09	0.09 ± 0.01
CEL1A	15.56 ± 1.36	8.35 ± 0.96	2.36 ± 0.29	0.96 ± 0.12	3.68 ± 0.21	2.38 ± 0.25	1.39 ± 0.23	2.34 ± 0.35	1.32 ± 0.12
CEL1B	0.23 ± 0.01	0.02 ± 0.00	–	–	–	0.68 ± 0.09	0.35 ± 0.07	0.05 ± 0.01	–

- Indicates not hydrolyzed. Values are the mean of three independent replicates

### Comparison of the catalytic properties of BGL3I with those of two intracellular β-glucosidases

**Fig. 5:**
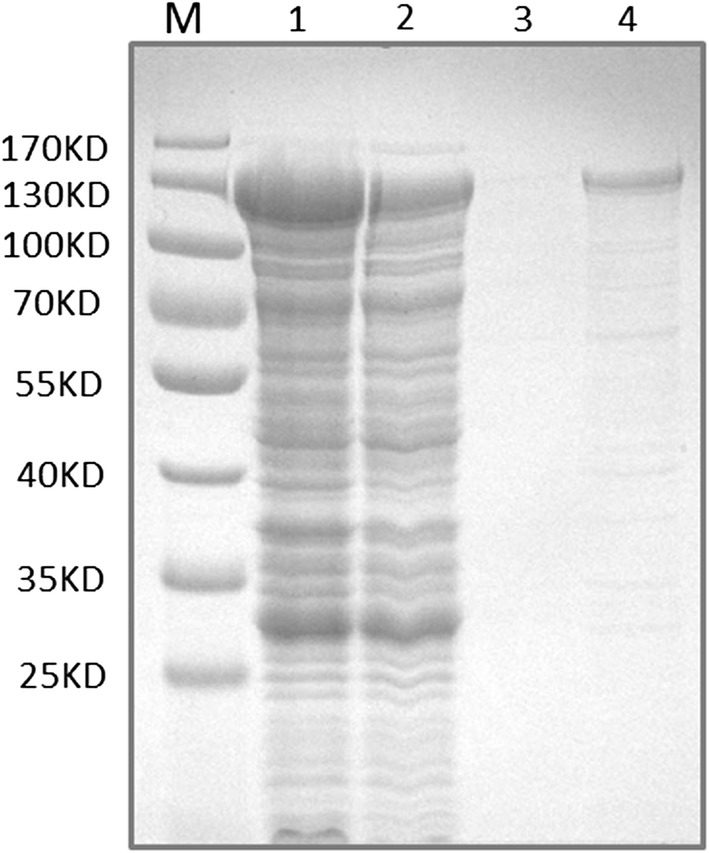
SDS-PAGE analysis of purified BGL3I. M, marker; 1, supernatant of lysate; 2, flow-through of discharged liquid; 3, collection of wash buffer; 4, eluate containing purified recombinant protein BGL3I-GST

BGL3I is predicted to be a secreted protein according to its signal peptide in the N-terminal (http://www.cbs.dtu.dk/services/SignalP/) [14]. However, using the constitutive promoter *pdc* or inducing promoters *cbh1* or *xyn2*, BGL3I was not detected in the fermentation supernatant by SDS-PAGE or Western blot when it was overexpressed in *T. reesei* Rut-C30. In contrast, BGL3I can be detected in the intracellular protein extract from the overexpression strains (Additional file 6).When using the constitutive promoter *tef1* (coding translation elongation factor 1), BGL3I was detected both in the fermentation supernatant and in the intracellular protein extract. However, it was obviously proteolyzed in the fermentation supernatant (Additional file 7). It indicates that BGL3I might serve as an intracellular protein functioning within cells rather than as an extracellular protein. To ensure whether most of the lactose taken up into the cells is hydrolyzed or transformed by BGL3I, both BGL3I and the main intracellular β-glucosidases, CEL1A and CEL1B, were heterologously expressed in *Escherichia coli* BL21 individually (Fig. 5) and their enzyme activities toward cellobiose (G2), cellotriose (G3), cellotetraose (G4), cellopentaose (G5), sophorose, lactose, salicin, *p*NPG, and ONPG were measured (Table 1). The purified BGL3I protein showed higher sophorose hydrolyzing activity than both CEL1A and CEL1B; however, it exhibited very weak activities toward all the other tested substrates (Table 1). CEL1A was the main intracellular β-glucosidase, and its ability to hydrolyze all substrates was much stronger than that of CEL1B (Table 1). The results indicate that BGL3I might hydrolyze sophorose, which was reported to be the most direct and strongest cellulase inducer, and was assumed to be the transglycosylation product of intracellular β-glucosidase [15–17].

**Fig. 6:**
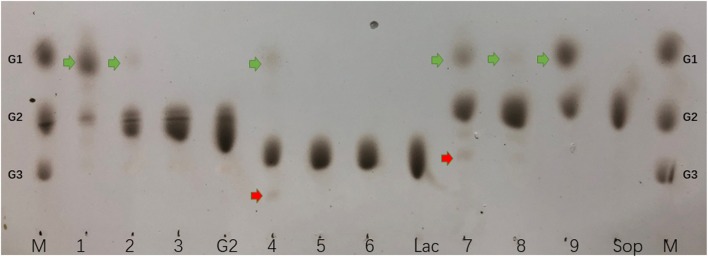
Thin-layer chromatography of cellobiose, lactose and sophorose hydrolysis by β-glucosidases. M: standard mixture of glucose (G1), cellobiose (G2), cellotriose (G3); 1: cellobiose + CEL1A; 2: cellobiose + CEL1B; 3: cellobiose + BGL3I; G2: cellobiose; 4: lactose + CEL1A; 5: lactose + CEL1B; 6: lactose + BGL3I; Lac: lactose; 7: sophorose + CEL1A; 8: sophorose + CEL1B; sophorose + BGL3I; Sop: sophorose. Green arrows: hydrolysis products; red arrows: transglycosylation products

Thin-layer chromatography was used to investigate the products of BGL3I, CEL1A and CEL1B using different disaccharides as substrates (1% cellobiose, 1% lactose, and 1% sophorose, respectively); the yields of the products from in vitro reactions were investigated at 50 °C for 1 h using equal quantities of protein. Thin-layer chromatography showed CEL1A hydrolyzed all three substrates into monosaccharide, and even CEL1B hydrolyzed the cellobiose and sophorose. However, BGL3I only hydrolyzed sophorose into monosaccharides. Moreover, transglycosylation activity of CEL1A was observed when lactose and sophorose were used as substrates (Fig. 6). However, when 1% cellobiose was used as the substrate, the transglycosylation products were not detected. Both CEL1B and BGL3I only hydrolyzed a small amount of lactose into monosaccharides. This result was in agreement with previously published enzyme activity assays [18].

### Comparison of the activity of the extracellular and intracellular proteins on sophorose hydrolysis

**Fig. 7:**
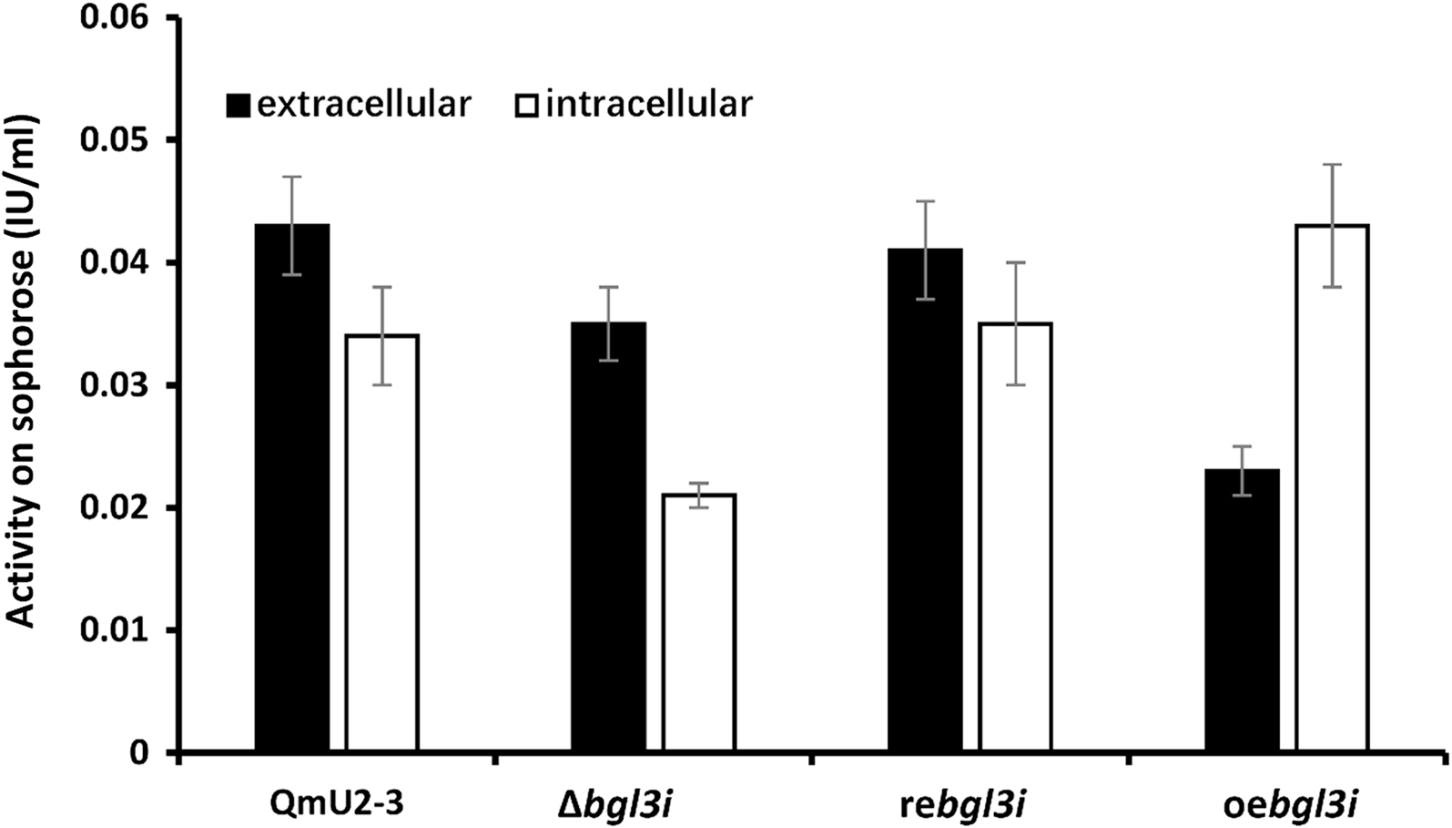
Bgl3I affected enzyme activity of extracellular and intracellular proteins on sophorose. QmU2–3: parent strain; Δ*bgl3i*: deletion strain, re*bgl3i*: complementation strain; oe*bgl3i*: overexpression strain. The enzyme activity of extracellular proteins is shown by solid bars and that of intracellular proteins is shown by hollow bars. Vertical bars indicate SD and each reaction was done in triplicate

Many extracellular and intracellular glycoside hydrolases belonging to the GH1 and GH3 families can hydrolyze sophorose, the strongest proposed natural inducer of cellulase synthesis [18]. Considering the complex composition of *T. reesei* cellulolytic enzymes, the enzyme activities of extracellular and intracellular proteins of the parent strain and the three progenies (deletion strain Δ*bgl3i*, complementation strain re*bgl3i*, and overexpression strain oe*bgl3i*) on sophorose were tested (Fig. 7). It showed the *bgl3i* deletion strain had the lowest intracellular activity on sophorose (0.021 ± 0.001 IU/ml), and the *bgl3i* overexpressed strain (under control of *tef1* promoter) had the highest activity (0.043 ± 0.005 IU/ml). It means that the decreased intracellular activity on sophorose hydrolysis in Δ*bgl3i* strain might provoke the cellulase synthesis and secretion. However, both extracellular activities on sophorose were decreased (*P *< 0.05) regardless of deletion or overexpression strain, compared with that of the parent strain or complementation strain. It indicated that the extracellular BGL3I did not provide the main activity on sophorose hydrolysis.

## Discussion

In total, 11 genes in *T. reesei* were annotated as β-glucosidase genes: *cel1a* (*bgl2*), *cel1b*, *cel3a* (*bgl1*), *cel3b*, *cel3c*, *cel3d*, *cel3e*, *cel3f*, *bgl3i* (*cel3* *g*), *cel3* *h,* and *bgl3j*. These genes were assigned to the GH1 family and GH3 family. The heterologously expressed products of these genes were purified from *E. coli* or *A. oryzae* and were characterized using different substrates [18]. Among the 11 genes, the functions of *cel3a*, *cel1a* and *cel1b* have been well studied and represent the secreted β-glucosidase and intracellular β-glucosidase, respectively. Deletion of *cel3a*, *cel1a* and *cel1b* could result in a decrease in the *xyr1* transcription level and cellulase production. All the three β-glucosidases were proved to provoke cellulase formation in *T. reesei*, but the mechanisms underlying it were different [19].

CEL3A is the major β-glucosidase in the enzyme preparation of *T. reesei*. It was reported that cellobiose usually accumulated in the cellulosic hydrolysate after hydrolysis by the *T. reesei* cellulase preparation, owing to its low β-glucosidase activity [20, 21]. Deletion of the *cel3a* gene in *T. reesei* resulted in delayed induction of cellulase genes [19, 22, 23], including a decrease in FPA and *p*NPG activities. In contrast, the introduction of a secreted heterologous GH3 family β-glucosidase from other fungi, or overexpression of CEL3A, could enhance the FPase activity and saccharification efficiency of the culture supernatant of *T. reesei* [20, 21]. The increased production of β-glucosidase might relieve the accumulation of cellobiose and thus its feedback-mediated inhibition of saccharification. It was hypothesized that CEL3A deletion resulted in accumulated cellobiose, which might in turn cause feedback-mediated inhibition of cellulase production or saccharification. CEL3B is another major β-glucosidase in *T. reesei*, which possesses similar enzyme activities to *p*NPG and other oligosaccharides such as CEL3A and has an even higher transcription level than CEL3A. However, until now there have been no reports addressing whether deletion of CEL3B may result in a similar phenotype as deletion of CEL3A [18, 24].

Similar to CEL3A, BGL3I was found to be a real β-glucosidase that cleaves β-1-4-bonds in cello-oligosaccharides into glucose units (Table 1); however, its ability to degrade *p*NPG and other oligosaccharides was relatively low compared with that of CEL3A. In addition, BGL3I was classified as a secreted β-glucosidase of the family GH3-like CEL3A [18]. However, BGL3I was not detected in the fermentation supernatant, being detected only in the intracellular protein extract even when it was overexpressed in *T. reesei* Rut-C30 (using the Ppdc constitutive promoter or the Pcbh1- and Pxyn2-inducing promoters; Additional file 6). The lack of BGL3I secretion might be due to its low protein concentration in *T. reesei*, or due to BGL3I being a real intracellular β-glucosidase, as previously annotated [25, 26]. CEL1A and CEL1B were the major intracellular β-glucosidases in *T. reesei*. CEL1A and CEL1B possess the highest and third highest transcription levels among the 11 annotated β-glucosidase in *T. reesei*, respectively [12]. CEL1A has relatively low *p*NPG activity, but high enzyme activities toward some oligosaccharides (Table 1). The deletion of CEL1A resulted in a significant delay in the transcription of *xyr1* and a delay in cellulase synthesis and secretion. CEL1B has the lowest enzyme activities to all of the test carbon sources (Table 1). However, the deletion of both CEL1A and CEL1B resulted in significantly decreased transcription of *xyr1* and cellulase synthesis [4]. CEL1A and CEL1B demonstrated synergistically regulated *xyr1* transcription and cellulase synthesis [4, 19]. It was previously reported that CEL1A was capable of catalyzing the transglycosylation of cellobiose to sophorose [17], which was reported to be the strongest inducer of cellulase synthesis in *T. reesei*. In our study, the transglycosylation ability of CEL1A was also confirmed (Table 1). Although BGL3I in *T. reesei* also influences *xyr1* transcription and cellulase induction, BGL3I did not show any transglycosylation ability. By contrast, it demonstrated high hydrolyzation of sophorose (Table 1).

Furthermore, the RPKM of *bgl3i* was the lowest among the 11 annotated β-glucosidase genes in Rut-C30 and its Δ*xyr1* strain [12]. A recent study reported another intracellular β-glucosidase with low RPKM in *T. reesei*, named CEL3D. The deletion of CEL3D also increased the cellulase activity, but had no effect on the expression levels of the main cellulase coding genes (such as *cel7a* and *cel7b*) [27]. In contrast, the deletion of *bgl3i* resulted in significantly increased extracellular protein production, as well as increased expression of cellulase coding genes and correlative TFs (Figs. 1, 2). CEL3D had average activity with *p*NPG, but almost no activity with several tested oligosaccharides, including laminaribiose and sophorose [18]. It is still unknown why and how the deletion of Cel3D affects the cellulase activity of *T. reesei* [27]; however, it is clear that the two low RPKM β-glucosidases, BGL3I and CEL3D, affect the cellulase activity of *T. reesei* according to different mechanisms. Understanding the molecular mechanism underlying the performance of BGL3I could help delineate the entire regulating system of cellulase production in *T. reesei.*

Although BGL3I was successfully heterologously expressed in *A.* *oryzae* [18], it was only detected in the intracellular protein extract in low quantities, even when it was overexpressed in *T. reesei* Rut-C30 using the Ppdc constitutive promoter, or Pcbh1- and Pxyn2-inducing promoters (Additional file 6). It seems that the transcription level of *bgl3i*, and the synthesis of BGL3I, in *T. reesei* might be strictly controlled. Conversely, in the Δ*bgl3i* strain, the expression levels of positive TFs involved in cellulase production, *xyr1* and *ace2*, were increased, especially after 16 h of induction. There was no significant difference in the transcription levels of the negative factors *cre1* and *ace1*. The phenotype of the Δ*bgl3i* strain was similar to that of the *T. reesei xyr1*-overexpressed strain [28]. It seems that the deletion of *bgl3i* reduced carbon catabolite repression or enhanced the induction of cellulase production in different carbon resources, including glucose (Additional file 3). However, it is unknown how BGL3I interacts with the TFs involved in cellulase production.

Both a previous study [18] and the present study demonstrated that BGL3I could cleave the β-1–2-bond in sophorose (Table 1), which is the most potent known inducer of cellulase in *T. reesei* and can be formed via transglycosylation of intracellular β-glucosidase [29]. BGL3I was only detected in the intracellular protein extraction, but not in the culture supernatant, when it was overexpressed in *T. reesei* (Additional file 6). These results suggest that BGL3I might hydrolyze the sophorose formed by intracellular β-glucosidase CEL1A or CEL1B, and that the decrease of sophorose might weaken cellulase induction in the parent strain. In contrast, deletion of BGL3I may ensure that the relatively high concentration of intracellular sophorose is maintained and thus strengthen the cellulase induction. This is the best explanation about why deletion of *bgl3i* increases *xyr1* transcription and cellulase induction.

**Fig. 8:**
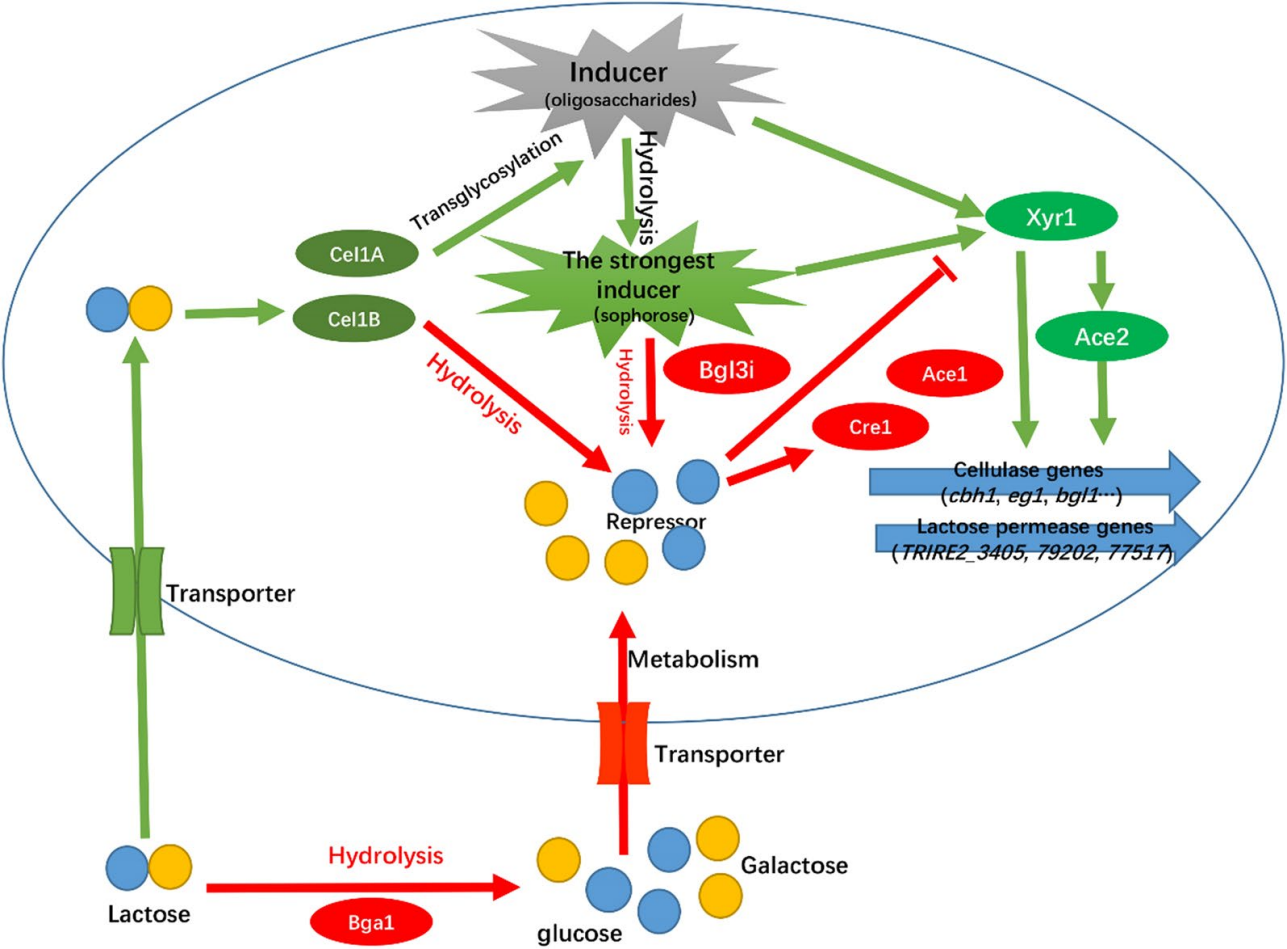
Model of a possible lactose induction process for initiating efficient cellulase formation

Despite extensive work related to the synthesis of cellulases in *T. reesei*, the real identity of the cellulase induction has not yet been established. It is known that *T. reesei* regulates cellulase production in response to sophorose [30] via a different mechanism compared with other lignocellulose-degrading fungi, including *P. oxalicum* [31, 32], *Neurospora crassa* [33] and *Aspergillus niger* [34]. Our study indicates that *T. reesei* may have subsequently retained the mycoparasitic characteristic for substrate competition, converting different saccharides into sophorose by a transglycosylation reaction, and then metabolizing sophorose [30]. This hypothesis is supported by the fact that new species-specific proteins were upregulated only in sophorose, and that cellobiose and sophorose were transported and metabolized at different rates [19]. Although the hydrolysis activity was limited, the transglycosylation activity preserved in a CEL1A mutant contributed to cellulase induction [17]. Furthermore, the accumulation of transglycosylation products (mainly sophorose) proved to be responsible for the increased cellulase activity [17]. This indicates that the transglycosylated products play important roles in cellulase induction. Based on the above data and analyses, it is proposed that BGL3I may hydrolyze the sophorose formed by transglycosylation and repress cellulase induction in *T. reesei* (Fig. 8).

## Conclusion

Due to its relatively high level of sophorose hydrolysis, BGL3I in *T. reesei* cells hydrolyzes sophorose formed by transglycosylation of lactose or cellobiose by CEL1A and thus represses the transcription of *xyr1* and different cellulase genes. By contrast, the deletion of *bgl3i* not only results in rapid uptake of lactose into the cell, which acts as the precursor of sophorose, but also decreases sophorose degradation. As a result, the intracellular sophorose remains relatively high in the Δ*bgl3i* strain, acting as an inducer for the transcription of TFs related to cellulase synthesis. BGL3I seems to be another molecular switch of cellulase induction in *T. reesei*. Taken together, *bgl3i* is a useful candidate gene for strain improvement. However, more research is still required to understand the mechanism underlying cellulase induction involving BGL3I and other β-glucosidases.

## Methods

### Microbial strains, plasmids and cultivation

*E. coli* DH5α was used as the cloning host. *Agrobacterium tumefaciens* AGL-1 was used as a T-DNA donor for fungal transformation [35]. *T. reesei* uridine auxotrophic *ura5*-negative strain QmU2–3, which was screened from mutants of QM9414 (ATCC 26921) by UV mutagenesis, served as the parent strain for *bgl3i* gene deletion. A T-DNA binary vector, NPxbthg, containing the selection marker gene *ura5* that encodes orotate phosphoribosyltransferase cloned from *P. oxalicum* under the control of the *Aspergillus nidulans gpdA* promoter and *trpC* terminator [36], was used for constructing the gene deletion mutant. To create *bgl3i*-overexpressed strain, we transformed *bgl3i* into QmU2–3 under control of the *cbh1* terminator in company with the *pdc* promoter, promoter *xyn2* and promoter *cbh1*, respectively. For complementation strain, the native *bgl3i* expression cassette was introduced into the *bgl3i* deletion strain to replace the *ura5* cassette.

Minimal medium with the addition of 300 μM cefotaxime and 0.2% Triton X-100 was used to select transformants, as described previously [37]. Each positive transformant was used to create mono-conidial cultures and the correct homologous integration was confirmed by PCR. All fungal strains were spread on PDA plates and grown at 28 °C for about 7 days for the conidial formation and then stored at 4 °C. The conidia of the fungal transformants were collected from PDA plates and inoculated into 50 ml flasks containing 10 ml SDB and cultured for 2 days at 28 °C with 200 rpm on a rotary shaker. Subsequently, mycelia were harvested and washed twice with minimal medium to remove the residual SDB. Equal amounts of mycelia were transferred into flasks with 10 ml minimal medium different carbon sources (1% lactose, cellobiose, Avicel, or glucose) and incubated at 28 °C with 200 rpm on a rotary shaker.

### Construction of *T. reesei bgl3i* deletion mutant

For *bgl3i* gene deletion, the 3050 bp *bgl3i* coding region was replaced by the selection marker gene *ura5* cassette. The non-coding regions ~ 1.5 kb upstream and downstream of the *bgl3i* gene were amplified from genomic DNA of WT using the primers shown in Additional file 8. The PCR fragment of the downstream non-coding region was digested with *Xho*I and then cloned into the *Xho*I site of NPxbthg using the ClonExpress™ One Step Cloning Kit (Vazyme Biotech, Nanjing, China), resulting in 3NPxbthg. Then, the PCR fragment of the upstream non-coding region was fused with the downstream non-coding region sequence, which was used for further marker-free digestion with *Bam*HI/*Hin*dIII and ligated into the corresponding site of vector NPxbthg, resulting in NPxbthg-*bgl3i*. The vector was transformed into the parent strain QmU2–3 by *A. tumefaciens*-mediated fungal transformation.

### Cellulase enzyme activity, protein concentration and biomass assay

For the cellulase enzyme activity assay, the culture supernatants of fungal strains were collected by centrifugation at 4 °C and 12,000×*g* for 10 min after being cultured for the indicated time period. The activity of endoglucanase, CMCase (substrate: CMC; Sangon, Shanghai, China), exo-1,4-β-glucanase, *p*NPCase (substrate: *p*NPC; Sigma, Saint Louis, MO, USA), and β-glucosidase, *p*NPGase (substrate: *p*NPG; Sigma, Saint Louis, MO, USA), and FPA, were assayed as described previously [20]. ONPG (Sigma, Saint Louis, MO, USA) served as the substrate for β-galactosidase activity assay, which was carried out in 100 μl reaction mixtures containing 50 μl substrate and 50 μl culture supernatant in 50 mM sodium acetate buffer (pH 5.0). The reaction mixtures were incubated at 50 °C for 10 min. The specific activities of β-glucosidases to cellobiose (G2: Sangon, Shanghai, China), cellotriose (G3: Sigma, Saint Louis, MO, USA), cellotetraose (G4: Sigma, Saint Louis, MO, USA), cellopentaose (G5: Sigma, Saint Louis, MO, USA), lactose (Sinopharm, Shanghai, China), and sophorose (Sigma, Saint Louis, MO, USA) were assayed using the Glucose Assay Kit (Sigma, Saint Louis, MO, USA). Mycelia were suspended in 50 mM sodium acetate buffer (pH 7.0) with protease inhibitor and homogenized by the FastPrep^®^-24 (MP Biomedicals, Solon, HO, USA) instrument. The supernatants of lysates were collected by centrifugation at 4 °C and 14,000×*g* for 10 min. Protein concentration was determined using a Bio-Rad protein assay kit (Bio-Rad, Hercules, CA, USA) according to the manufacturer’s instructions. The biomass assay was performed as described previously [38].

TLC was used to investigate the products of BGL3I, CEL1A, and CEL1B using different disaccharides as substrates (1% cellobiose, 1% lactose, and 1% sophorose, respectively); the yields of the substrate products from each in vitro reactions (200 μl) were investigated at 50 °C for 1 h using equal (50 μg) quantities of protein. The compositions of the hydrolysates were analyzed by TLC, and ethyl acetate–isopropanol–water (3:3:1, v/v/v) was used as the developing solution. The spots were visualized by soaking the plates in methanol containing 20% (v/v) sulfuric acid, followed by incubation at 110 °C for 10 min.

### Lactose concentration determination

Lactose concentration was determined using DNS [39] with lactose as the standard. The standard curve was created using gradient lactose concentrations varying from 0 to 10 mg/ml at intervals of 2 mg/ml. The determination of residual lactose in the culture supernatant was carried out in 200 μl reaction mixtures containing 80 μl culture supernatant and 120 μl DNS. The reaction mixtures were incubated at 95 °C for 10 min and then 40 μl of each sample was mixed with 160 ddH_2_O in a microplate (Greiner Bio-One, Frickenhausen, Germany). The data of OD_540_ were collected.

### RNA extraction, reverse transcription and quantitative RT-PCR

Mycelia were harvested for the indicated time period after cultivation and transferred into TRIzol reagent (Invitrogen, Carlsbad, CA, USA). Two biological replicates were prepared in the process. RNA extraction, reverse transcription, and quantitative RT-PCR were performed as reported previously [40]. The primers used in RT-PCR are described in Additional file 8.

### Heterologous expression of recombinant BGL3I in *E. coli*

The BGL3I coding sequence was expressed by the pGEX system according to the manufacturer’s guidelines. The first-strand cDNA was used as a template to amplify the fragment encompassing the ORF using the primers indicated in Additional file 2: Table S1. The fragment was then ligated into plasmid pGEX-4T-1 (GE Healthcare, Piscataway, NJ, USA) via *Bam*HI and *Xho*I double digestion to produce pGEX-4T-*bgl3i*, and was subsequently introduced into *E. coli* BL21 (DE3) for protein production [41]. The recombinant protein was purified using a GST-Bind resin column (Merck KGaA, Darmstadt, Germany) from the lysate of cells according to the supplier’s recommendations. PBS (50 mM NaH_2_PO_4_, 150 mM NaCl) with 1 mM phenylmethylsulfonyl fluoride (Sangon, Shanghai, China), 1 mM dithiothreitol (Sangon, Shanghai, China), 1 mM ethylenediaminetetraacetic acid (Sangon, Shanghai, China), and 1% (v/v) Triton X-100 (Sinopharm, Shanghai, China) was used for the lysis buffer. PBS with 1 mM dithiothreitol and 1 mM ethylenediaminetetraacetic acid was used as the wash buffer. Elution buffer [50 mM Tris (pH 8.0), 0.4 M NaCl, 50 mM l-glutathione reduced (Sigma, Saint Louis, MO, USA), 0.1% Triton X-100, 1 mM dithiothreitol] was used to collect the GST-tagged BGL3I. SDS-PAGE was used to verify the purified protein according to standard protocols (120 V, 90 min) [42]. The GST moiety was removed by thrombin (1 U/mg of fusion protein; Novagen, Madison, WI, USA) on the column and incubation for 16 h at 4 °C. Analysis of protein concentration was performed using the Bradford Protein Assay kit (Sangon, Shanghai, China).

### Statistical analyses

Student’s *t* test was performed with WPS Office software (Kingsoft Corp., Beijing, China), employing a two-tailed test. Significance was at *P* < 0.05.

## Additional files

**Additional file 1.** Deletion of *bgl3i* gene from QmU2–3 derived strain. (A) Schematic map of replacing the *bgl3i* coding region with marker *ura5* gene by the homologous integration, generating *bgl3i* deletion strain; (B) Identification of positive deletion strain by PCR: M, marker 1 kb ladder; +, positive control; -, negative control.

**Additional file 2.** Protein concentration and enzyme activities of QmU2–3 derived strain. QmU2–3: parent strain; Δ*bgl3i*: *bgl3i* deletion strain; re*bgl3i*: complementation strain. Samples were supernatants of each strain after 7-day incubation with 1% lactose.

**Additional file 3.** Cellulase activity of *T. reesei* on other carbon source. (A) CMCase activity, (B) *p*NPCase activity, (C) *p*NPGase activity, (D) PFU activity, (E) extracellular protein concentration of WT and Δ*bgl3i* mutant on 1% cellobiose, Avicel or glucose. Vertical bars indicate SD and each reaction was done in triplicate.

**Additional file 4.** Transcriptional levels of the major cellulase genes response to Avicel. (A) *cbh1*, (B) *egl1*, (C) *bgl1*. Cellulase genes were detected 4 h, 16 h and 24 h after the beginning of the induction on 1% Avicel in WT and Δ*bgl3i* mutant. Transcripts were normalized to the housekeeping gene *actin*. Vertical bars indicate SD and each reaction was done in triplicate.

**Additional file 5.** Analysis of transcription levels of transcription factors. (A) transcription factor *xyr1*, (B) transcription factor *cre1*, (C) transcription factor *ace1*, (D) transcription factor *ace2*. They were detected 4 h, 16 h and 24 h after the beginning of the cultivation on 1% Avicel in strains WT and Δ*bgl3i* mutant. Transcripts were normalized to the housekeeping gene *actin*. Vertical bars indicate SD and each reaction was done in triplicate.

**Additional file 6.** Western blot analysis of BGL3I-his overexpressed in *T. reesei* QmU2–3 using promoter *pdc*, *xyn2* and *cbh1*. Western blot analysis of BGL3I-his in the intracellular protein extract (A) and in the culture supernatant (B) of BGL3I-his overexpressed transformants. Lane 1: transformant of *bgl3i* overexpressed under control of promoter *pdc*; lane 2: transformant of *bgl3i* overexpressed under control of promoter *xyn2*; lane 2: transformant of *bgl3i* overexpressed under control of promoter *cbh1*; M: marker.

**Additional file 7.** Western blot analysis of BGL3I-his overexpressed in *T. reesei* QmU2–3 using constitutive promoter *tef1*. Western blot analysis of BGL3I-his in the intracellular protein extract (A) and in the culture supernatant (B) of BGL3I-his overexpressed transformant. Lane 1: sample cultured for 5 days; lane 2: sample cultured for 7 days; M: marker.

**Additional file 8.** Oligonucleotides used in this study.

## Abbreviations

GH: glycoside hydrolase
SDS-PAGE: sodium dodecyl sulfate-polyacrylamide gel electrophoresis
SDB: Sabouraud Dextrose Broth
PDA: potato dextrose agar
WT: wild type
FPA: filter paper activity
CMC: carboxymethylcellulose sodium
*p*NPC: *p*-nitrophenol-d-cellobioside
*p*NPG: *p*-nitrophenol-d-glucopyranoside
*O*NPG: *O*-nitrophenyl-d-galactopyranoside
DNS: dinitrosalicylic acid
JGI: Joint Genome Institute
RPKM: reads per kilobase million
TF: transcription factor
ORF: open reading frame
TLC: thin-layer chromatography
PBS: phosphate buffer saline
