# Supplementary figures and images for: The putative β-glucosidase BGL3I regulates cellulase induction in Trichoderma reesei
Source: Biotechnol Biofuels. 2018 Nov 19;11:314. doi: 10.1186/s13068-018-1314-6 (PMC6240962; doi:10.1186/s13068-018-1314-6)

**
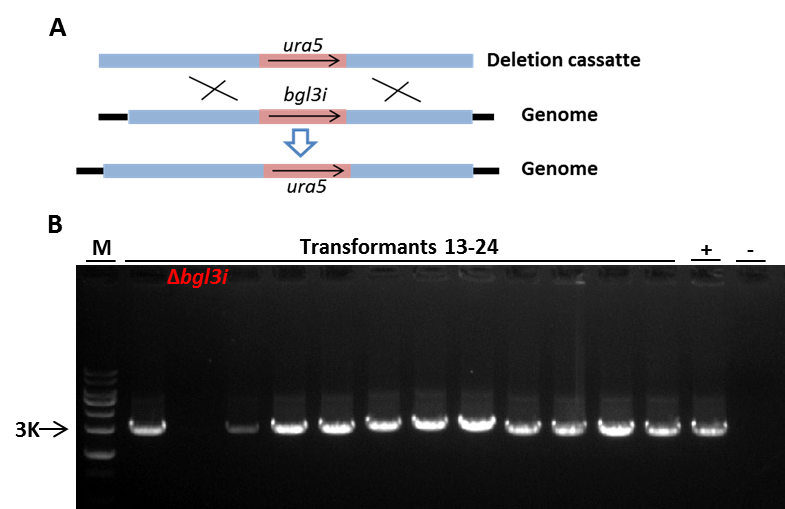
**

Supplement: Supplementary file 1 — Additional file 1. Deletion of bgl3i gene from QmU2–3 derived strain. (A) Schematic map of replacing the bgl3i coding region with marker ura5 gene by the homologous integration, generating bgl3i deletion strain; (B) Identification of positive deletion strain by PCR: M, marker 1 kb ladder; +, positive control; -, negative control. [file 13068_2018_1314_MOESM1_ESM.docx]

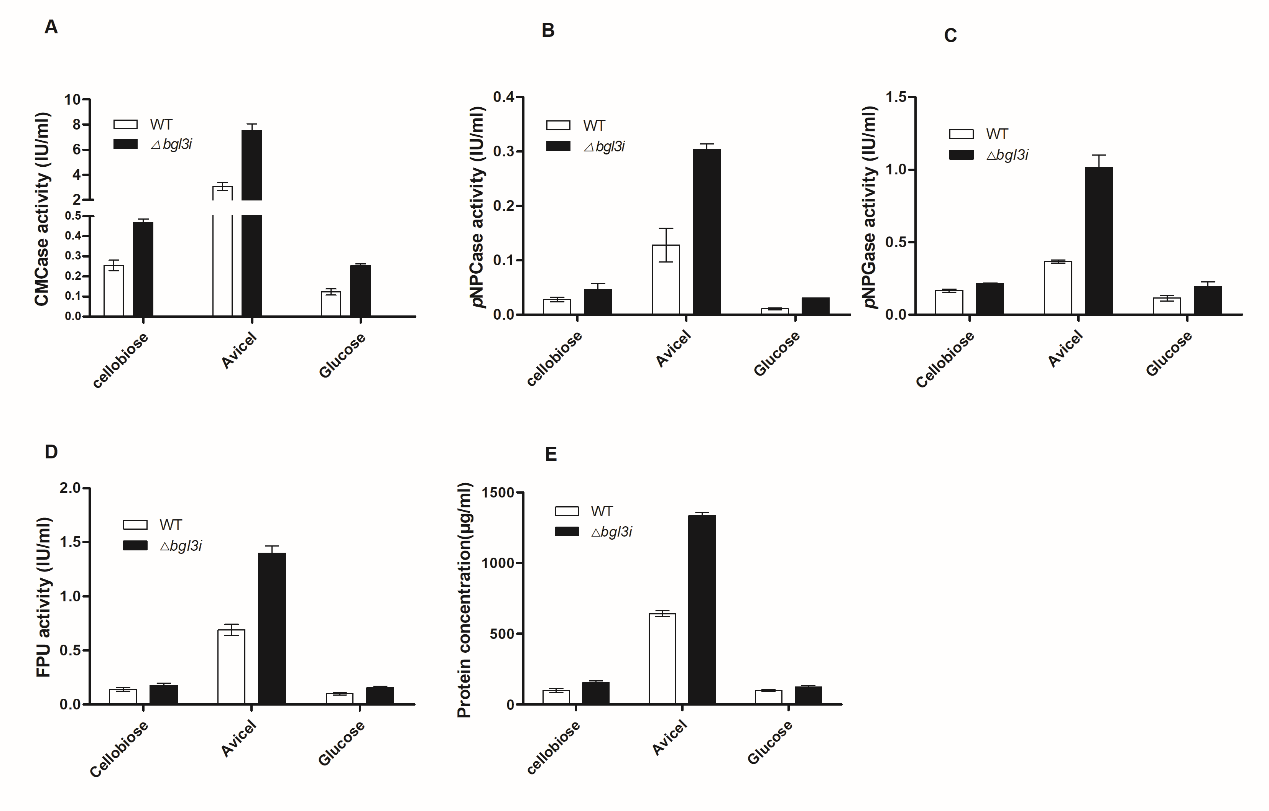

Supplement: Supplementary file 3 — Additional file 3. Cellulase activity of T. reesei on other carbon source. (A) CMCase activity, (B) pNPCase activity, (C) pNPGase activity, (D) PFU activity, (E) extracellular protein concentration of WT and Δbgl3i mutant on 1% cellobiose, Avicel or glucose. Vertical bars indicate SD and each reaction was done in triplicate. [file 13068_2018_1314_MOESM3_ESM.docx]

**
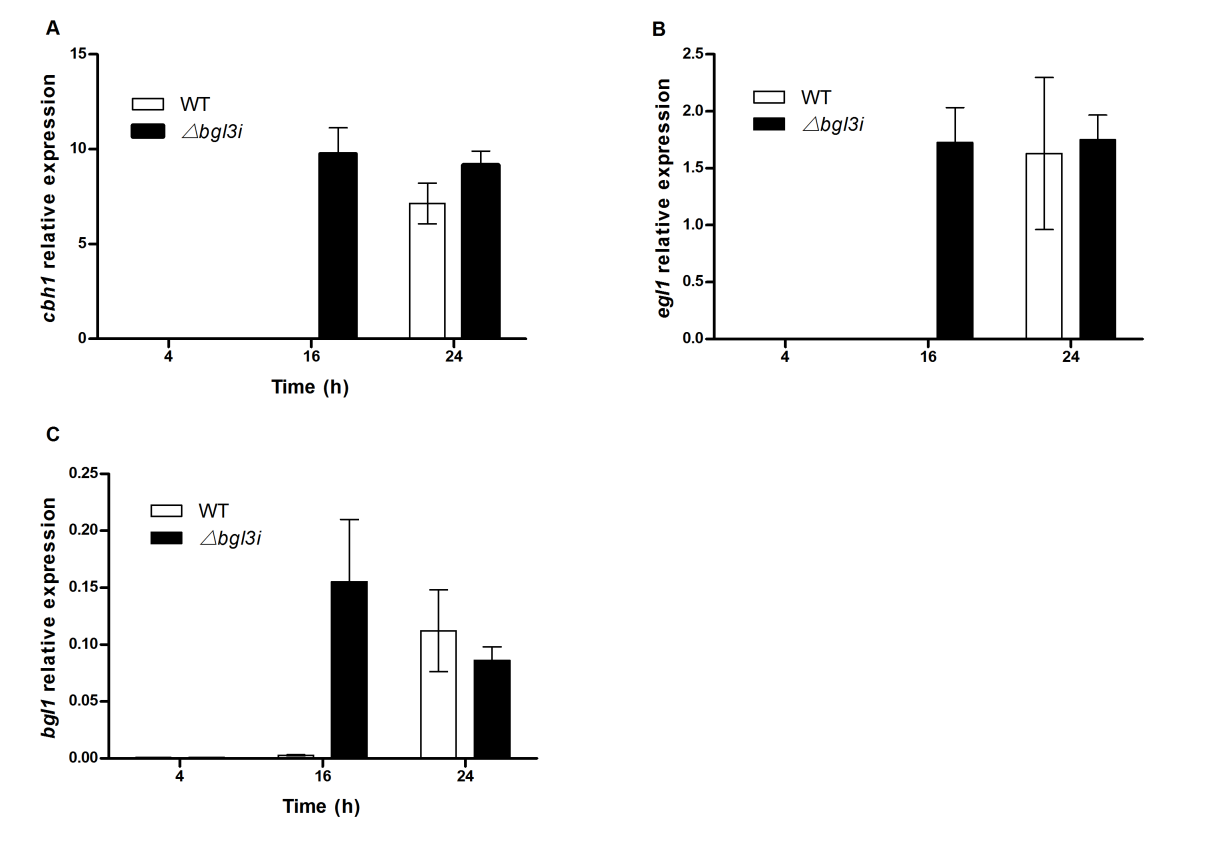
**

Supplement: Supplementary file 4 — Additional file 4. Transcriptional levels of the major cellulase genes response to Avicel. (A) cbh1, (B) egl1, (C) bgl1. Cellulase genes were detected 4 h, 16 h and 24 h after the beginning of the induction on 1% Avicel in WT and Δbgl3i mutant. Transcripts were normalized to the housekeeping gene actin. Vertical bars indicate SD and each reaction was done in triplicate. [file 13068_2018_1314_MOESM4_ESM.docx]

**
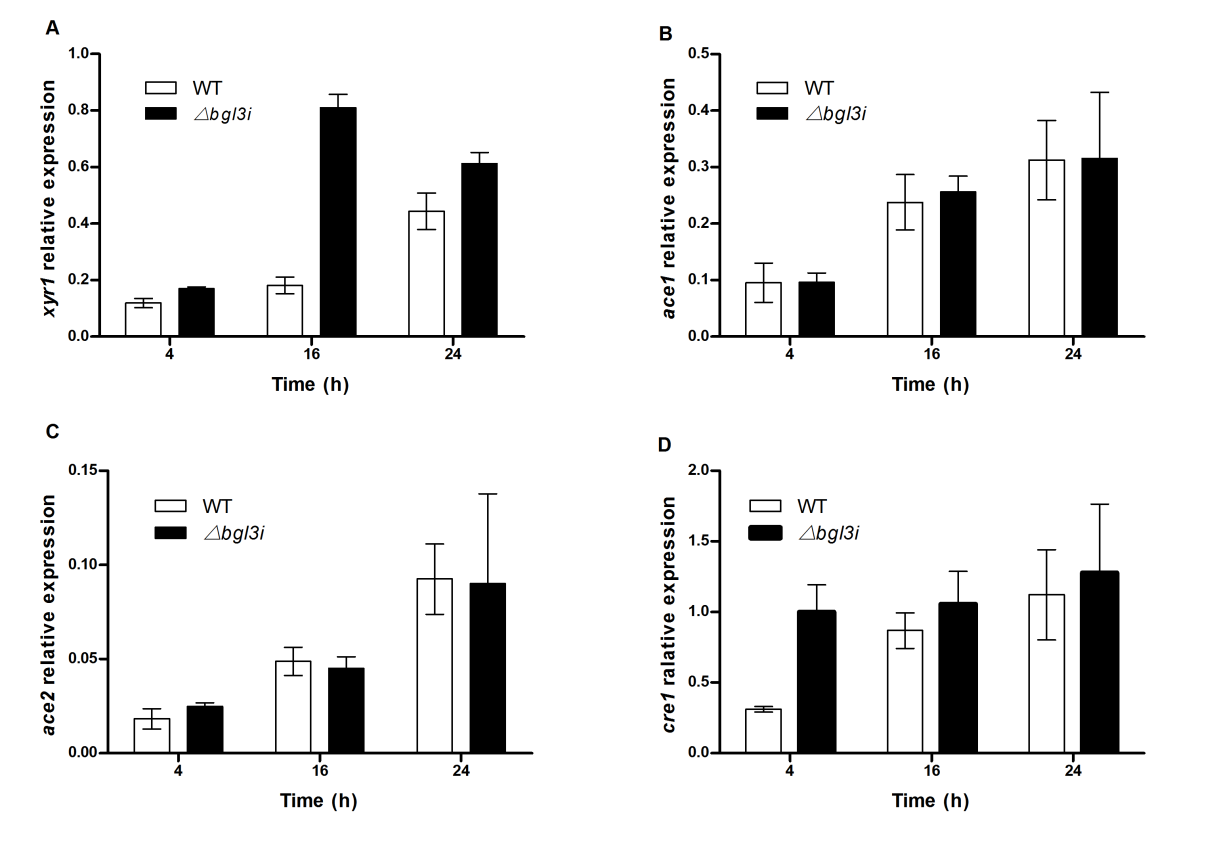
**

Supplement: Supplementary file 5 — Additional file 5. Analysis of transcription levels of transcription factors. (A) transcription factor xyr1, (B) transcription factor cre1, (C) transcription factor ace1, (D) transcription factor ace2. They were detected 4 h, 16 h and 24 h after the beginning of the cultivation on 1% Avicel in strains WT and Δbgl3i mutant. Transcripts were normalized to the housekeeping gene actin. Vertical bars indicate SD and each reaction was done in triplicate. [file 13068_2018_1314_MOESM5_ESM.docx]

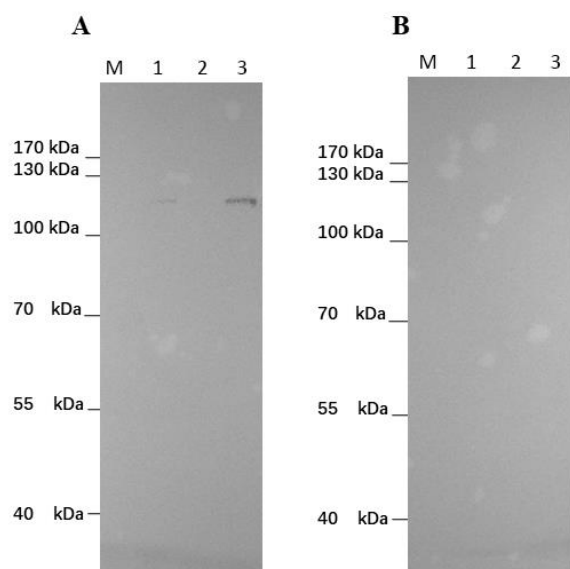

Supplement: Supplementary file 6 — Additional file 6. Western blot analysis of BGL3I-his overexpressed in T. reesei QmU2–3 using promoter pdc, xyn2 and cbh1. Western blot analysis of BGL3I-his in the intracellular protein extract (A) and in the culture supernatant (B) of BGL3I-his overexpressed transformants. Lane 1: transformant of bgl3i overexpressed under control of promoter pdc; lane 2: transformant of bgl3i overexpressed under control of promoter xyn2; lane 2: transformant of bgl3i overexpressed under control of promoter cbh1; M: marker. [file 13068_2018_1314_MOESM6_ESM.pdf]

**A**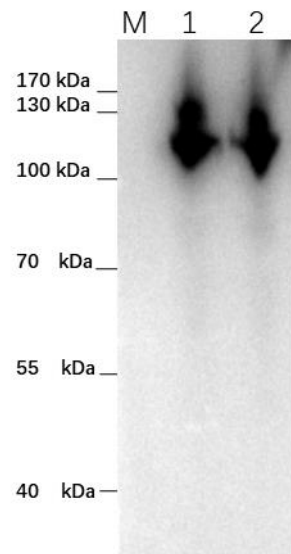**B**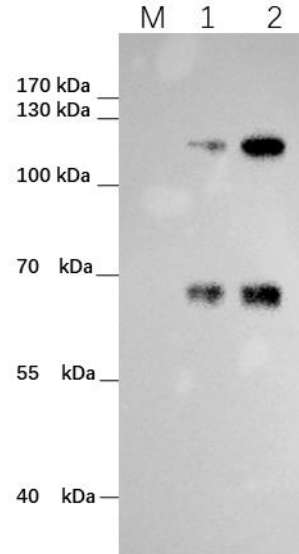

Supplement: Supplementary file 7 — Additional file 7. Western blot analysis of BGL3I-his overexpressed in T. reesei QmU2–3 using constitutive promoter tef1. Western blot analysis of BGL3I-his in the intracellular protein extract (A) and in the culture supernatant (B) of BGL3I-his overexpressed transformant. Lane 1: sample cultured for 5 days; lane 2: sample cultured for 7 days; M: marker. [file 13068_2018_1314_MOESM7_ESM.pdf]
