# Supplementary material for: The putative β-glucosidase BGL3I regulates cellulase induction in Trichoderma reesei
Source: Biotechnol Biofuels. 2018 Nov 19;11:314. doi: 10.1186/s13068-018-1314-6 (PMC6240962; doi:10.1186/s13068-018-1314-6)
Supplement: Supplementary file 2 — Additional file 2. Protein concentration and enzyme activities of QmU2–3 derived strain. QmU2–3: parent strain; Δbgl3i: bgl3i deletion strain; rebgl3i: complementation strain. Samples were supernatants of each strain after 7-day incubation with 1% lactose. [file 13068_2018_1314_MOESM2_ESM.docx]

**Table S1 Protein concentration and enzyme activities of QmU2-3 derived strain**

| **strain** | **QmU2-3** | **Δ*bgl3i*** | **re*bgl3i*** | **oe*bgl3i*** |
| --- | --- | --- | --- | --- |
| **Protein Conc. (mg/ml)** | **0.216±0.005** | **0.280±0.02** | **0.218±0.009** | **0.147±0.011** |
| **CMCase (IU/ml)** | **0.533±0.042** | **1.602±0.081** | **0.491±0.087** | **0.311±0.043** |
| ***p*NPGase(IU/ml)** | **0.075±0.011** | **0.158±0.041** | **0.069±0.012** | **0.041±0.009** |
| ***p*NPCase(IU/ml)** | **0.008±0.001** | **0.035±0.001** | **0.008±0.000** | **0.005±0.000** |
| **FPA(IU/ml)** | **0.208±0.014** | **0.267±0.013** | **0.212±0.022** | **0.141±0.012** |
