# Supplementary material for: The putative β-glucosidase BGL3I regulates cellulase induction in Trichoderma reesei
Source: Biotechnol Biofuels. 2018 Nov 19;11:314. doi: 10.1186/s13068-018-1314-6 (PMC6240962; doi:10.1186/s13068-018-1314-6)
Supplement: Supplementary file 8 — Additional file 8. Oligonucleotides used in this study. [file 13068_2018_1314_MOESM8_ESM.docx]

**Oligonucleotides used in this study**

| Primers | Sequences (5 '–3 ')_a_ | Notes |
| --- | --- | --- |
| bgl3i5F | CCCAAGCTTATCAACAGCCAGCAGCCATC | For *bgl3i* gene deletion and confirmation |
| bgl3i5R | CGGGATCCCGTGCAGAAGCGGAGACACG |  |
| bgl3i3F | ATTCCGTCACCAGCCCTGCTCGAGGTAGGTATATGTCTGATTAGC |  |
| bgl3i3R | CACACATTATTATGGAGAAACTCGAGGATCGATATTGGGATACATACG |  |
| bgl3iFF | TGTCTGTTTATAATCTGGGCCATTGGCGTCGTAGGTATATGTCTGATTAGC |  |
| bgl3iFR | GCATGCTAATCAGACATATACCTACGACGCCAATGGCCCAGATTATAAAC |  |
| bgl3iYF | ATGACCTCGTTTCACGACG |  |
| bgl3iYR | CTACACAGGCCCAACAAC |  |
| Bgl3iF1 | ACCTCGTTTCACGACGGCGTCAAGTTGTCCACTGTGACGTATGGCTACTATGTGCCGC | For Bgl3i-GST expression and purification |
| Bgl3iR1 | CACTCACCCATATAGATGCC |  |
| Bgl3iF2 | CATCTATATGGGTGAGTGGCGATGTGCCGGAAACAC |  |
| Bgl3iR2 | CAATATTTGAGCTGTATGCGTATTG |  |
| Bgl3iF3 | CGCATACAGCTCAAATATTGACGATAGGACGCTGCATGAAG |  |
| Bgl3iR3 | GAGCACGCAGTCCCGTTCACAGCATTGTAAGCCATCATGACG |  |
| Bgl3iF4 | GTGAACGGGACTGCGTGCTC |  |
| Bgl3iF | CTGGTTCCGCGTGGATCCACCTCGTTTCACGACGGCGT |  |
| Bgl3iR | CTCGAGTCGACCCGGGAATTCCTACACAGGCCCAACAACACCAG |  |
| actinF | TCCATCATGAAGTGCGAC | For quantitative RT-PCR |
| actinR | GTAGAAGGAGCAAGAGCAGTG |  |
| xyr1F | CCATCAACCTTCTAGACGAC |  |
| xyr1R | AACCCTGCAGGAGATAGAC |  |
| cre1F | AGGCACGCCAAGAGGTCAA |  |
| cre1R | GGACAGGTTTCTCAGACTCGG |  |
| ace1F | GGACGAGGAGGAGATTATG |  |
| ace1R | GTGAGTCTTCTCGTGCTT |  |
| ace2F | GACAAGAAGCTCAGGTGTC |  |
| ace2R | ACTGTGTTCATGGCTGTG |  |
| cbh1F | CTTGGCAACGAGTTCTCTT |  |
| cbh1R | TGTTGGTGGGATACTTGCT |  |
| egl1F | CGGCTACAAAAGCTACTACG |  |
| egl1R | CTGGTACTTGCGGGTGAT |  |
| bgl1F | AGTGACAGCTTCAGCGAG |  |
| bgl1R | GGAGAGGCGTGAGTAGTTG |  |
| cel1aF | CGTGCTCTTCACCAACAA |  |
| cel1aR | TCTTGCTGATCCACACCA |  |
| cel1bF | CGATTTCCAAGGCCATT |  |
| cel1bR | TTGAGGGTGGTGTAGTCTGT |  |
| 3405F | CATGGACAAGATTGGCTG |  |
| 3405R | TCGTCAAGCTCTTCCAAA |  |
| 77517F | ATCTGGGTGGCGTTCATCTT |  |
| 77517R | TCGTCTCGTTGCTGGATCTC |  |
| 79202F | TCGGAGTGATGAATCTCGTTTT |  |
| 79202R | CGTCGCCTCCTTGTTTCTC |  |
